# Towards a hand-held, fast, and sensitive gas chromatograph-ion mobility spectrometer for detecting volatile compounds

**DOI:** 10.1007/s00216-020-03059-9

**Published:** 2020-11-21

**Authors:** André Ahrens, Stefan Zimmermann

**Affiliations:** Institute of Electrical Engineering and Measurement Technology, Department of Sensors and Measurement Technology, Leibniz University Hannover, Appelstr. 9A, 30167 Hannover, Germany

**Keywords:** Ion mobility spectrometer, IMS, Gas chromatograph, GC, Bundled GC columns, Hand-held GC-IMS

## Abstract

Ion mobility spectrometers can detect gaseous compounds at atmospheric pressure in the range of parts per trillion within a second. Due to their fast response times, high sensitivity, and limited instrumental effort, they are used in a variety of applications, especially as mobile or hand-held devices. However, most real-life samples are gas mixtures, which can pose a challenge for IMS with atmospheric pressure chemical ionization mainly due to competing gas-phase ionization processes. Therefore, we present a miniaturized drift tube IMS coupled to a compact gas chromatograph for pre-separation, built of seven bundled standard GC columns (Rtx-Volatiles, Restek GmbH) with 250 μm ID and 1.07 m in length. Such pre-separation significantly reduces chemical cross sensitivities caused by competing gas-phase ionization processes and adds orthogonality. Our miniaturized GC-IMS system is characterized with alcohols, halocarbons, and ketones as model substances, reaching detection limits down to 70 ppt_v_ with IMS averaging times of just 125 ms. It separates test mixtures of ketones and halocarbons within 180 s and 50 s, respectively. The IMS has a short drift length of 40.6 mm and reaches a high resolving power of *R*_P_ = 68.

**Figure:**
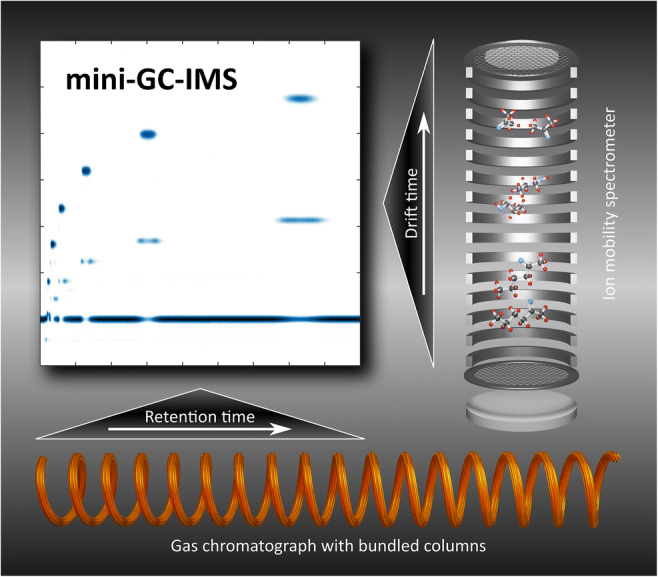
Graphical abstract

Graphical abstract

## Introduction

Drift tube ion mobility spectrometers (IMS) often consist of three axially arranged regions: the reaction, drift, and detector regions. Once a gaseous sample has been ionized in the reaction region by an ionization source, the ions are shifted into the drift region through an ion gate. A neutral drift gas flow at atmospheric pressure continuously flushes the drift region. Here, driven by a homogeneous electric field, the ions are separated on their way along the axis of the drift region based on their ion mobility. The low field ion mobility is defined as the proportionality factor between the mean drift velocity and the electric drift field at low reduced electric drift fields [1]. Low field conditions are typically given for reduced electric fields between 2 and 10 Td depending on the ion species [2]. The low field ion mobility can be determined by the length of the drift region *L*, the drift voltage *U*_d_, and the drift time *t*_d_. However, since the low field ion mobility depends on the ion-neutral pair collision cross section and the ion mass, often the reduced ion mobility *K*_0_ is given for comparable results [1, 3]. Here, the influence of a changing number of neutral gas particles due to temperature or pressure changes is eliminated by normalizing to standard temperature *T*_0_ = 273.15 K and the standard pressure *p*_0_ = 1013.25 hPa, as shown in Eq. (1).

1 $$ {K}_0=\frac{L^2}{t_{\mathrm{d}}\cdotp {U}_{\mathrm{d}}}\cdotp \frac{T_0}{T}\cdotp \frac{p}{p_0} $$

At the end of the drift region after several milliseconds of drift time, the ions discharge at a detector, often a Faraday plate. Recording the ion current over time results in an ion mobility spectrum, which contains information about the sample gas composition. Thus, IMS can rapidly detect volatile and semi-volatile substances down to the ppt_v_ level (parts per trillion) within a second [4]. The detection limits are given according to the 3*σ* definition. Technically, the detection limit is a calculated concentration giving a signal three times higher than the standard deviation of the noise at zero concentration.

In the past, IMS have gained in importance as powerful instruments for monitoring, detecting, and identifying compounds in air. Applications are in the field of safety and security, forensics, life sciences, and food quality, where complex sample mixtures can often occur [5–12]. For some of these applications, mobile or hand-held devices are beneficial. Consequently, system miniaturization is necessary. Different approaches for miniaturized IMS drift tubes have been presented, still showing fast response times and low detection limits [13–17]. However, gas mixtures always pose a challenge for IMS not only due to the limited resolving power but moreover due to chemical cross sensitivities associated with atmospheric pressure chemical ionization (APCI). The ion population does usually not reflect the composition of a gas mixture, up to the case of complete suppression of substances with low proton or electron affinity in the presence of substances with high proton or electron affinity. Thus, limited IMS resolving power is often not the main issue, but the reaction chemistry of APCI. To solve this issue, a gas chromatograph (GC) is often coupled to IMS for pre-separation [18, 19]. This also adds orthogonality for increased separation power and clear substance identification by both retention time and drift time. The approach of GC-IMS has been first investigated in 1970 by Cohen et al. [20]. Later, it was also transferred to portable systems [21, 22]. In GC, a carrier gas (mobile phase) transports a gaseous sample volume through a glass or stainless steel capillary (column) with the inner capillary wall coated with a thin liquid film (stationary phase, unpacked column). Due to interactions with the stationary phase, the individual compounds of the mixture are retained differently as they pass through the capillary and therefore elute at different retention times *t*_R_. Thus, by coupling a GC to an IMS, chemical cross sensitivities in the IMS reaction region can be significantly reduced and orthogonality is added. However, co-eluting substances from a GC might not be further separated by orthogonal IMS, since in extreme cases only one of the co-eluting substances is ionized, while the other substances are fully suppressed. From this point of view, the IMS converts to a GC detector, but an extremely sensitive one. Nevertheless, even if the GC alone can solve the separation task, an IMS with highest possible resolving power is needed to detect a substance peak close to the reactant ion peak (RIP) always present in IMS with APCI. Thus, increasing IMS resolving power increases the spectrum of detectable compounds. Furthermore, many co-eluting substances can be ionized simultaneously in IMS and can thus be further separated orthogonally, but quantification is difficult if not impossible.

Commercially available standard columns vary in inner diameters (ID) between 0.15 and 0.53 mm, whereas the film thickness ranges from 0.1 up to 10 μm. Without delving deeply into the complex theory of gas chromatography, one relevant aspect is important for miniaturizing GC. For short retention times, as required for hand-held devices with sufficient separation power as well as carrier gas flow rates suitable for IMS, several columns in parallel with small inner diameter are beneficial [23].

In this work, we present a compact GC-IMS, consisting of a miniaturized high-performance drift tube IMS and a small GC, built from seven bundled commercially available standard GC columns (Rtx-Volatiles, Restek GmbH) with 250 μm ID and 1.07 m in length. Due to the small overall dimensions of 170 mm × 110 mm × 55 mm, it fits the requirements for integration into hand-held devices for sensitive and rapid detection of volatile compounds.

## Experimental

**Fig. 1:**
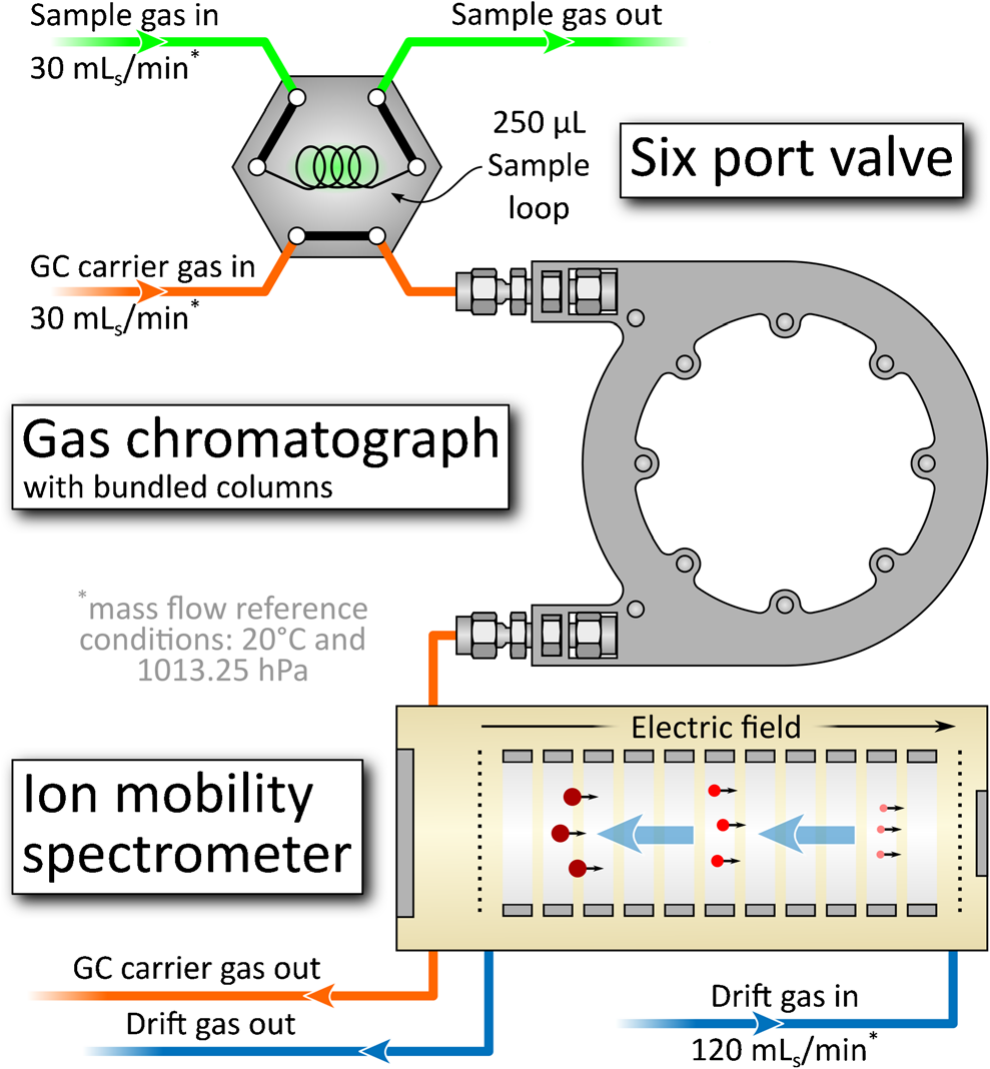
Scheme of the GC-IMS. A six-port valve is used as sample inlet. Subsequently, a GC with bundled columns and a drift tube IMS are used in series. The GC pre-separates the sample and thus avoids competing ionization processes in the IMS reaction region. Furthermore, it adds orthogonality

The developed GC-IMS consists of three main components: a six-port valve with a 250-μL sample loop, a GC, and a drift tube IMS. A scheme of the setup is shown in Fig. 1.

The 250-μL sample loop is continuously flushed by a 30 mL_s_/min flow (mass flow reference conditions 20 °C and 1013.25 hPa) of the sample gas (Fig. 1, green line). On demand, the six-port valve (MTV-6SL-N32UF-1 by Takasago Electric Inc., Japan) shifts the sample loop into the constant 30 mL_s_/min GC carrier gas flow (Fig. 1, orange line). This way, a sample gas volume of 250 μL is injected into the GC. The GC consists of seven bundled standard GC columns (Rtx-Volatiles by Restek GmbH, Germany) with 250 μm ID and 1.07 m in length. Although 30 mL_s_/min (4.3 mL_s_/min per column) GC carrier gas flow is high, here, it is a good compromise between separation performance and separation time, as the latter should be short for high repetition rates in hand-held applications such as the detection of hazardous compounds. For all, sample gas, GC carrier gas, and IMS drift gas, purified dry (dew point < − 90 °C) air is used since additional gas cylinders for nitrogen or helium, which are regularly used as GC carrier gases, are not an option for hand-held devices. Nevertheless, air as GC carrier gas limits the maximum GC temperature to about 80 °C. Higher temperatures can lead to a damage of the stationary phase, when using oxygen containing carrier gases. The volume of the reaction region of the IMS is just 160 μL keeping the GC resolving power. The IMS used in this work has been basically described elsewhere [24]. Limits of detection have been determined in the range of ppt_v_ within averaging times of 1 s. Outer overall dimensions are only 15 mm × 15 mm × 56 mm. A slight modification to the design described in [24] has been made by adding a second waste capillary (Fig. 1, orange line, GC carrier gas out) directly connected to the reaction chamber to obtain better purging of this region. Detailed operating parameters of the IMS are given in Table 1. The resolving power of the used IMS is *R*_P_ = 68.

**Table 1:** Dimensions, gases, and operating parameters of the GC-IMS system

Parameters	Value
Sample loop volume	250 μL
Sample loop gas flow	30 mL_s_/min
Sample gas	Air (purified)
GC carrier gas flow	30 mL_s_/min
GC carrier gas	Air (purified)
IMS drift gas flow	120 mL_s_ /min
IMS drift gas	Air (purified)
Dew point of drift, sample and GC carrier gas	− 90 °C
IMS ionization source	Tritium
IMS ionization source activity	130 MBq
IMS ionization source active diameter	10 mm
Field switching injection field	282.5 V/mm
Field switching blocking field	− 0.25 V/mm and 0.45 V/mm
Drift length *L*	40.6 mm
Drift region diameter	8 mm
Drift region field	63.5 V/mm
Repetition rate	80 Hz
GC column length	1.07 m
Dew point of drift gas and sample gas	− 90 °C
GC and sample loop temperature	40 °C
IMS temperature *T*	38 °C
Inner IMS pressure *p*	1001–1019.5 hPa

**Fig. 2:**
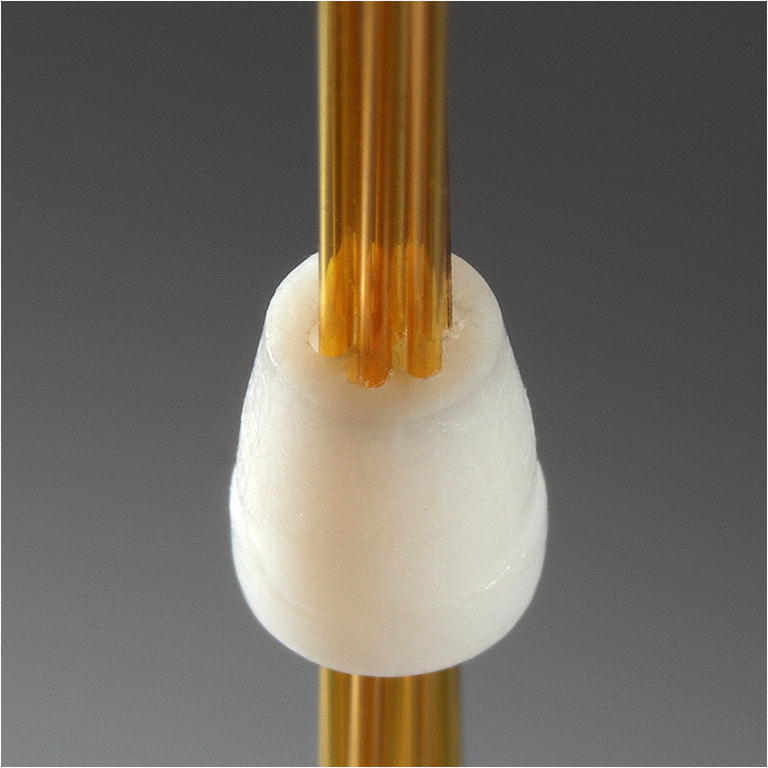
Photo of the modified 1/16″ PTFE ferrule with seven 0.5-mm bores and 250-μm-ID standard fused silica GC columns inserted (Rtx-Volatiles, Restek GmbH, Germany)

In parallel operation of the GC columns, alignment of the single columns to each other is crucial for the analytical performance. Thus, commercially available 1/16″ no-hole PTFE ferrules (No. T1600 by BGB Analytik Vertriebs GmbH, Germany) are modified with seven 0.5 mm bores, as shown in Fig. 2, to align and seal each GC column without any adhesive.

**Fig. 3:**
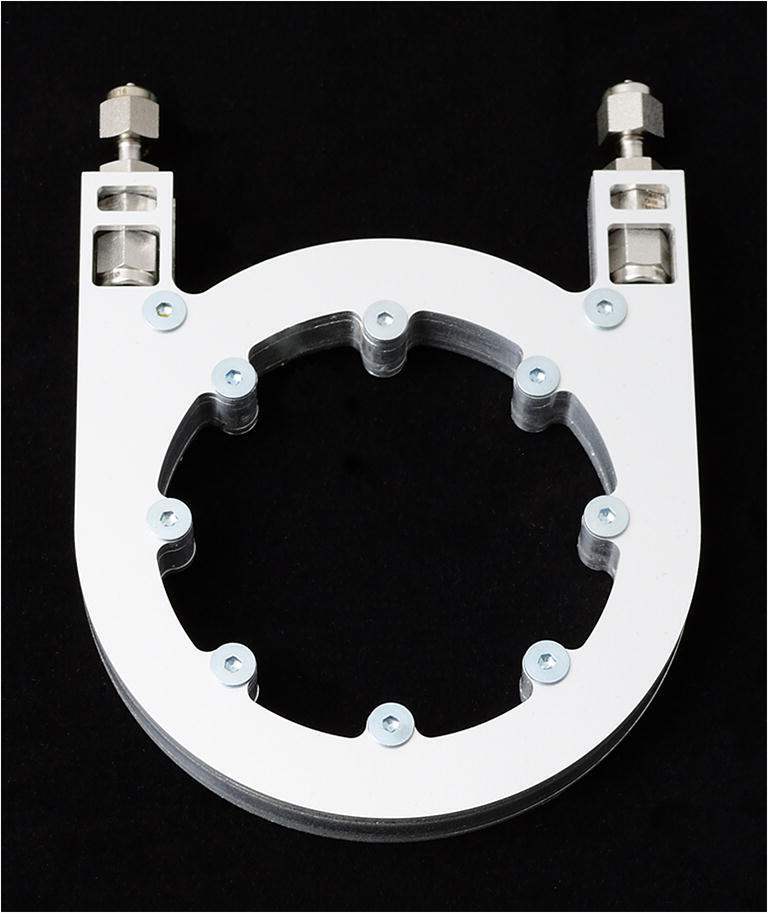
Photo of the small aluminum GC “oven” (80 mm × 105 mm × 11 mm (width × length × height)) with 1/16″ stainless steel bulkhead unions (top) for easy connection

Subsequently, through bore 1/16″ stainless steel bulkhead unions are mounted at the ends of the column bundle. To protect the individual GC columns from mechanical stress during operation and to ensure a homogenous temperature of the GC, the bundle is placed into a small GC “oven.” The oven design is based on layered aluminum sheets. In the middle of the rectangular cross section of the oven, a 4 mm × 4 mm channel is milled to house the bundled GC columns. To ensure homogeneous heat distribution, four smaller channels are milled equidistant from the center channel to keep an electrically insulated heating wire (NiCr 80/20, 0.7 mm diameter). The design also offers GC temperature profiles, but this would cause additional energy consumption for heating not desired for a hand-held device. Furthermore, no active cooling is implemented also for energy reasons. Flow profiles are also possible, but not considered due to the increased energy consumption associated with higher pumping rates. The GC oven with its small dimensions of only 80 mm × 105 mm × 11 mm and a weight of 95 g is shown in Fig. 3. This small and light weight design is easy-to-integrate into the closed-loop IMS demonstrator described in [24]. In this context, it needs to be mentioned that this demonstrator and in particular the periphery (filters, pumps, electronics, battery, etc.) are not size optimized compared to a future product close to market.

Still keeping low energy consumption in mind, as necessary for long operating times of a battery-powered hand-held devices, the GC is operated isothermally at 40 °C. This way, energy consumption of the GC oven is kept at 1.7 W in steady state at 20 °C ambient temperature. Since condensation of eluents in the transfer line between the GC and the IMS must be avoided, the six-port valve seat, sample loop, GC, and IMS drift tube are all placed together in a small, thermally insulated housing with outer dimensions of only 170 mm × 110 mm × 55 mm. Inside this housing, the waste heat of the GC oven is evenly distributed by small fans, so that the IMS, six-port valve, and sample loop are also kept close to 40 °C. To prevent overheating, the solenoid of the six-port valve is placed outside the housing.

An overview of all dimensions, gases, and operating parameters of the GC-IMS system is shown in Table 1.

## Results and discussion

As a benchmark, a mixture of seven ketones (acetone, 2-butanone, 2-pentanone, 2-hexanone, 2-heptanone, 2-octanone, and 2-nonanone) in positive ion mode of the IMS and a mixture of three halocarbons (1,2-dichloropropane, 1,1,2-trichloroethane, and 1,2,3-trichloropropane) in negative ion mode have been investigated.

**Fig. 4:**
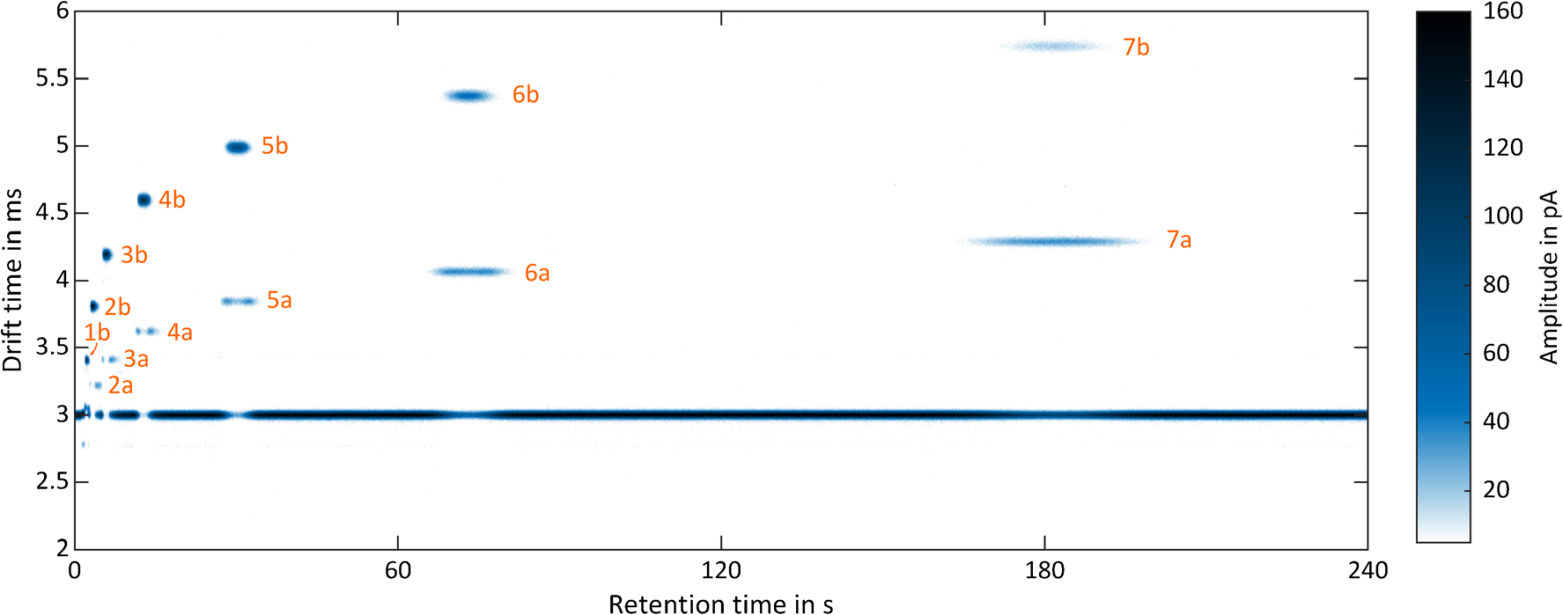
GC-IMS heat map of a ketone mixture (each substance at 500 ppb_v_) of (1) acetone, (2) 2-butanone, (3) 2-pentanone, (4) 2-hexanone, (5) 2-heptanone, (6) 2-octanone, and (7) 2-nonanone of in purified, dry air. Suffixes: monomers (-a) and dimers (-b). Operating parameters are given in Table 1. IMS pressure *p* = 1019.5 hPa

**Fig. 5:**
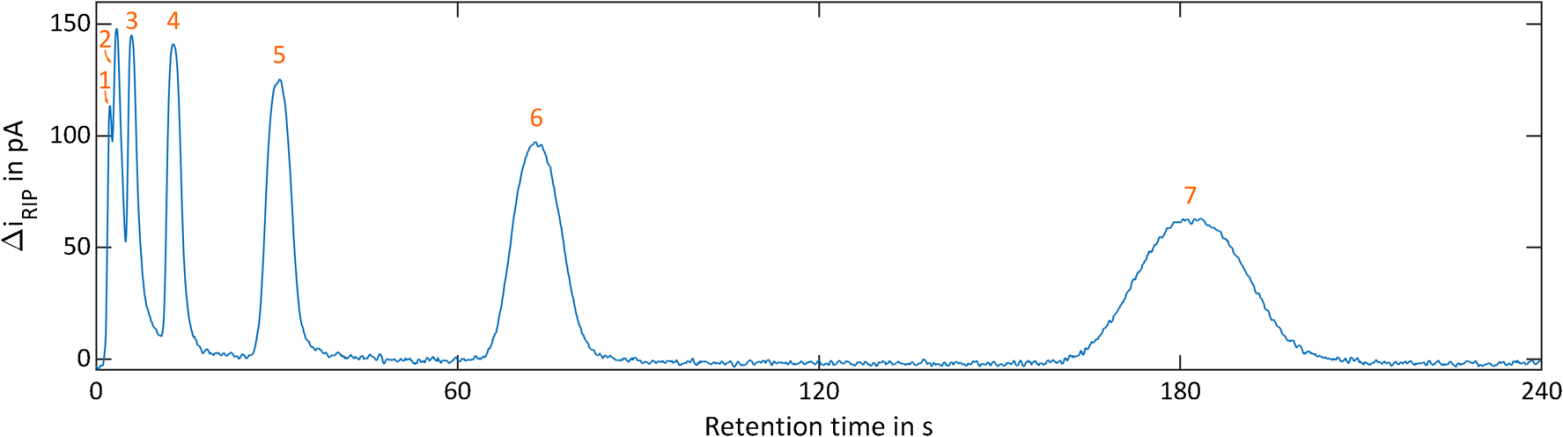
Chromatogram of a ketone mixture (each substance at 500 ppb_v_) of (1) acetone, (2) 2-butanone, (3) 2-pentanone, (4) 2-hexanone, (5) 2-heptanone, (6) 2-octanone, and (7) 2-nonanone in purified, dry air. Operating parameters are given in Table 1. IMS pressure *p* = 1019.5 hPa

Due to the temporal pre-separation by the GC, the measurement results from the GC-IMS are two-dimensional. The first dimension is given by the retention time of the GC; the second dimension is the drift time of the analyte ions inside the IMS. IMS spectra are continuously recorded during the GC run. Thus, data are typically presented in heat maps. For data acquisition, a self-developed software in LabView was used. For visualization and data processing, MATLAB by MathWorks and OriginPro by OriginLabs were used. Figure 4 shows the measurement results of the ketone mixture with 500 ppb_v_ per substance in dry, purified air. Each number (1–7) represents one compound: (1) acetone, (2) 2-butanone, (3) 2-pentanone, (4) 2-hexanone, (5) 2-heptanone, (6) 2-octanone, (7) 2-nonanone. Monomers and dimers can be differentiated by the suffixes -a and -b, respectively. The “continuous” signal of the IMS at a drift time of *t*_d_ =3 ms is the positive reaction ion peak (RIP), caused by continuous ionization of the GC carrier gas. If an analyte elutes from the GC, the reactant ions ionize the analyte, and thus, the RIP decreases. A chromatogram can be obtained, when plotting the RIP amplitude over time, as shown in Fig. 5. As can be seen, the GC alone cannot fully resolve the mixture while the GC-IMS can.

In the case of acetone, the RIP partly covers the monomer peak under dry conditions. Thus, the acetone monomer appears as a shoulder of the RIP in Fig. 4 leading to a reduced signal 1 in Fig. 5, which precludes the determination of the detection limit for the acetone monomer. Furthermore, it can be observed that isothermal operation of the GC with constant flow leads to peak broadening and increased retention times. Comparing the heat map in Fig. 4 with the chromatogram in Fig. 5, the mixture is clearly separated in about 3 min by the GC-IMS due to its increased orthogonality.

**Fig. 6:**
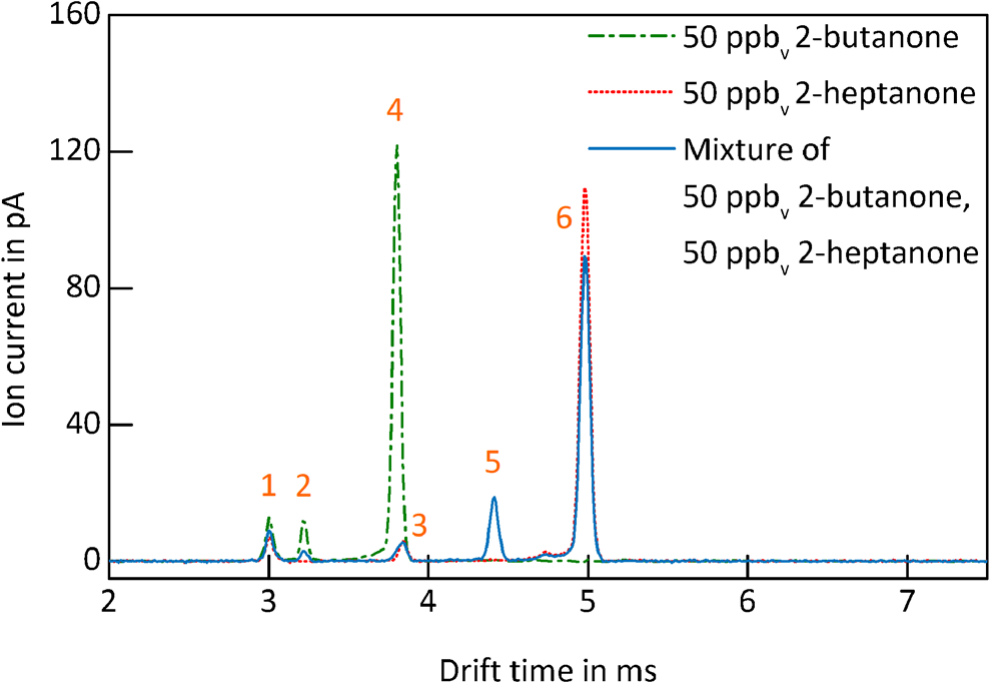
Ion mobility spectra of 50 ppb_v_ 2-butanone (green, dashed dotted line), 50 ppb_v_ 2-heptanone (red, dotted line), and a mixture of 50 ppb_v_ 2-butanone and 50 ppb_v_ 2-heptanone (blue, continuous line) in purified, dry air. Operating parameters are given in Table 1. IMS pressure *p* = 1020 hPa

As mentioned above, mixtures are always challenging for IMS, not only due to the limited IMS resolving power but moreover due to the competing ionization processes in the IMS reaction region, which can lead to strong discrimination of low proton or electron affine compounds. This makes quantification and even detection of volatile compounds in mixtures difficult if not impossible. To demonstrate the benefit of GC pre-separation in this context, 50 ppb_v_ 2-butanone, 50 ppb_v_ 2-heptanone, and a mixture of 50 ppb_v_ 2-butanone and 50 ppb_v_ 2-heptanone in purified, dry air are analyzed by the IMS alone. Since the 2-butanone dimer and the 2-heptanone monomer have similar drift times (see Fig. 4), both peaks cannot be separated by the IMS alone. The obtained ion mobility spectra are shown in Fig. 6.

**Fig. 7:**
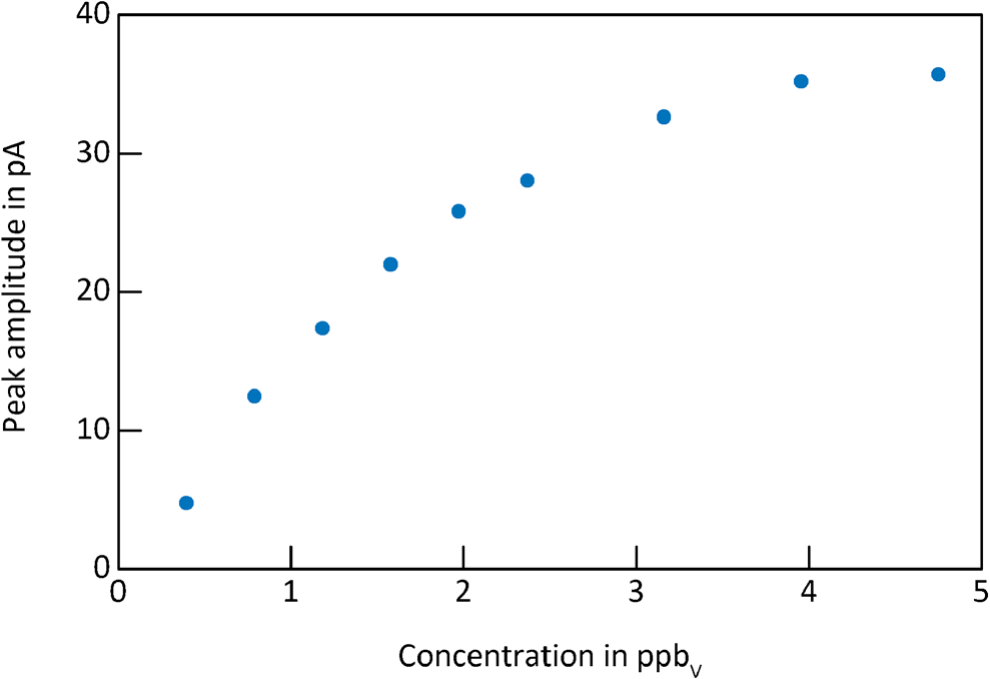
GC-IMS calibration curve of 2-butanone monomer in purified, dry air. Operating parameters are given in Table 1. IMS pressure *p* = 1014.5 hPa

The ion mobility spectrum of 50 ppb_v_ 2-butanone (green, dashed dotted line) shows a residue of the RIP (peak 1), a tiny monomer peak (peak 2), and a large dimer peak (peak 4). The ion mobility spectrum of 50 ppb_v_ 2-heptanone (red, dotted line) shows a residue of the RIP (peak 1); a tiny monomer, covered by peak 3; and a large dimer peak (peak 6). The ion mobility spectrum of the mixture (blue, continuous line) shows both a spectral overlap of the 2-butanone dimer and the 2-heptanone monomer (broadened peak 3) but even more important a strong suppression of the 2-butanone signals due to the presence of 2-heptanone. Furthermore, peak 5 is probably a mixed dimer caused by ion-molecule reactions. As shown in Fig. 4, with pre-separation, the mixed dimer peak cannot form and the discrimination effect is significantly reduced demonstrating the benefit of coupling a GC to IMS. Figure 7 shows a typical GC-IMS calibration curve for monomers, here for the 2-butanone monomer, used to determine the limits of detection.

**Fig. 8:**
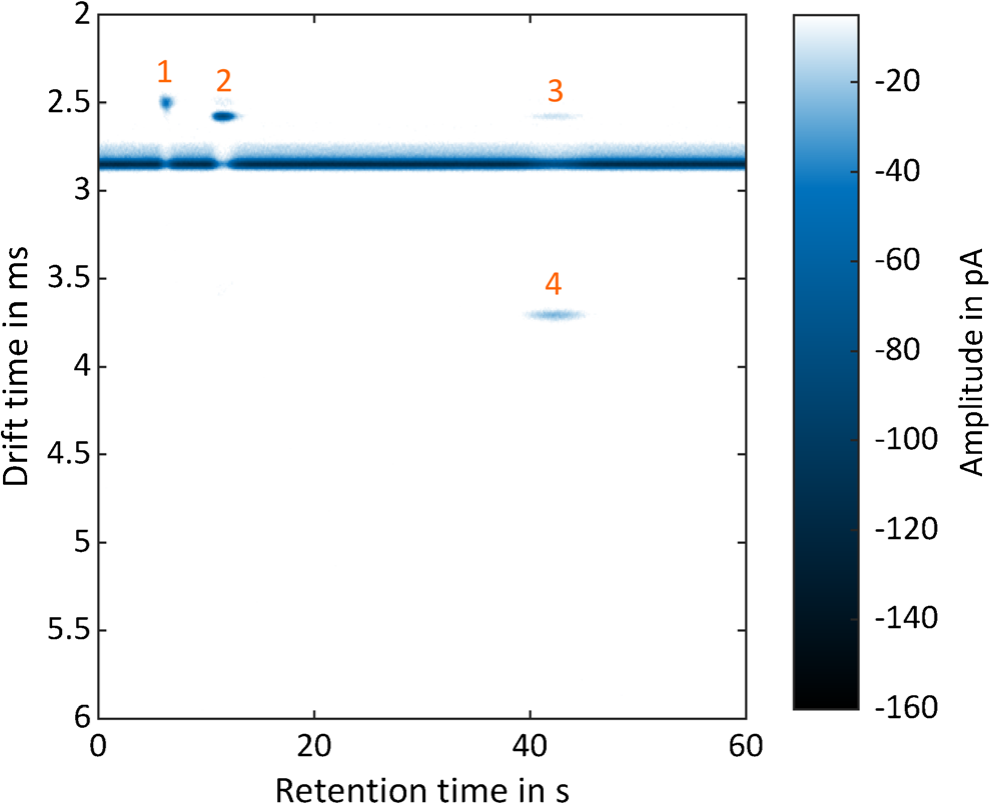
GC-IMS heat map of a halocarbon mixture of (1) 1 ppm_v_ 1,2-dichloropropane, (2) 138 ppb_v_ 1,1,2-trichloroethane, and (3, 4) 41 ppb_v_ 1,2,3-trichloropropane. Peaks 1, 2, and 3 are potential fragments of the initial compounds containing the chlorine. Operating parameters are given in Table 1. IMS pressure *p* = 1012 hPa

For demonstrating the negative ion mode, a mixture of 1 ppm_v_ 1,2-dichloropropane, 138 ppb_v_ 1,1,2-trichloroethane, and 41 ppb_v_ 1,2,3-trichloropropane has been investigated. The corresponding heat map is shown in Fig. 8. Due to the chemical characteristics of the substances, it can be assumed that the measured peaks with shorter drift times than the negative RIP at *t*_*d*_ = 2.85 ms are fragments of the respective compounds containing the chlorine.

In addition to the above test mixtures, alcohols have been investigated. Retention times, reduced ion mobilities and detection limits of all investigated substances are listed in Table 2. Regarding the differences in detection limits when comparing stand-alone IMS [24] and GC-IMS, the following aspects should be kept in mind besides dilution effects due to peak broadening. Dilution effects in fittings and in the IMS reaction region, although the reaction region was kept at a minimum. Furthermore, due to the short retention times and peak widths for the volatile compounds in this work, IMS averaging times are kept at 125 ms, to capture all compounds eluting from the bundled GC. Often, IMS detection limits are given for averaging times of 1 s, instead of 125 ms as given here. Longer averaging times lead to a reduced noise level, better signal to noise ratio, and, thus, improved detection limits.

**Table 2:** Retention time, reduced ion mobility, and detection limit of different ketones, alcohols, and halocarbons measured with the GC-IMS setup. Operating parameters are given in Table 1.

Compound	CAS number	Retention time *t*_R_ in s	Polarity	Reduced ion mobility *K*_0_ in cm^2^/(Vs)	Detection limit GC-IMS in ppb_v_ within 125 ms averaging time	Reduced ion mobility *K*_0_ in cm^2^/(Vs)	Detection limit GC-IMS in ppb_v_ within 125 ms averaging time
				Monomer		Dimer	
Reactant ion peaks							
Positive RIP	-	n.a.	Positive	2.11	-	-	-
Dominant negative RIP	-	n.a.	Negative	2.19	-	-	-
Ketones							
Acetone	67-64-1	2.3	Positive	1.97	-	1.77	1.65
2-Butanone	78-93-3	3.4	Positive	1.87	0.27	1.58	2.00
2-Pentanone	107-87-9	5.9	Positive	1.76	0.28	1.44	2.20
2-Hexanone	591-78-6	12.8	Positive	1.66	0.53	1.31	3.76
2-Heptanone	110-43-0	30.3	Positive	1.57	1.13	1.21	6.11
2-Octanone	111-13-7	73.3	Positive	1.48	2.21	1.12	14.80
2-Nonanone	821-55-6	182.2	Positive	1.40	3.47	1.05	26.26
Alcohols							
Ethanol	64-17-5	1.9	Positive	1.93	0.37	1.76	6.34
1-Propanol	71-23-8	2.5	Positive	1.80	0.29	1.57	5.41
2-Propanol	67-63-0	2.0	Positive	1.83	0.07	1.62	1.05
1-Butanol	71-36-3	4.2	Positive	1.69	0.11	1.42	1.78
tert-Butanol	75-65-0	2.2	Positive	1.74	0.26	1.50	3.23
3-Methyl-1-butanol	123-51-3	7.2	Positive	1.60	0.11	1.31	1.53
1-Pentanol	71-41-0	9.5	Positive	1.59	0.58	1.30	4.84
Halocarbons							
1,2-Dichloroproane	78-87-5	6.3	Negative	2.38	88.00	-	-
1,1,2-Trichloroethane	79-00-5	11.6	Negative	2.32	2.85	-	-
1,2,3-Trichloropropane	96-18-4	42.3	Negative	1.61	10.13	-	-

When comparing our results with commercially available GC-IMS, it should be noted that our work shows preliminary results of a first demonstrator meant to be integrated into future hand-held devices. Commercially available GC-IMS are mainly benchtop instruments, e.g., manufactured by G.A.S. Gesellschaft für analytische Sensorsysteme mbH, Germany; ION-GAS GmbH, Germany; or MaSa Tech, s.r.o., Slovakia, where the IONdrug instrument from ION-GAS is the smallest device with 300 mm × 300 mm × 160 mm. Other systems worth mentioning are the benchtop GC-IONSCAN by Smiths Detection [18] and a hand-held prototype by Graseby [21, 22], which has much lower IMS resolving power of just *R*_P_ = 10 to 25 but larger dimensions compared to our miniaturized IMS. The IMS used in the benchtop systems have higher or similar analytical performance but are difficult to integrate in hand-held devices. The GC used in the benchtop systems have higher separation power but longer GC columns and use temperature and flow profiles, which makes a comparison difficult. Due to longer retention times and higher power consumption, integration into hand-held devices and rapid detection of, e.g., hazardous volatile compounds are difficult. Thus, compared to other GC-IMS, the setup presented here outperforms in relation to size. A device comparable in size is the Dräger X-PID, a hand-held GC-PID, with a multi-capillary GC having similar resolving power compared to our GC, but much higher limits of detection, due to the photo ionization detector.

## Conclusion

In this work, a miniaturized GC-IMS for integration into future hand-held devices for fast and sensitive detection of volatiles is presented. Coupling a compact GC for pre-separation to our miniaturized IMS not only adds orthogonality but also significantly reduces chemical cross sensitivities when measuring gas mixtures. The GC is built from seven bundled standard GC columns (Rtx-Volatiles, Restek GmbH) with 250 μm ID and 1.07 m in length for short retention times, sufficient separation power to reduce chemical cross sensitivities, and enough sample flow rate compatible with IMS. Avoiding gas cylinders not suitable in hand-held applications, air is used as GC carrier gas, which limits the maximum temperature of the GC and thus, its usability for analysis of semi-volatile compounds. As a benchmark, detection limits for volatiles as alcohols, halocarbons, and ketones are determined and mixtures of ketones and halocarbons have been investigated. The detection limits range from 70 ppt_v_ (2-propanol) to 88 ppb_v_ (1,2-dichloroproanes) for averaging times of 125 ms in isothermal operation at 40 °C. The ketone mixture and the halocarbon mixture were completely separated by the miniaturized GC-IMS within 180 s and 50 s, respectively.

## References

[1] Eiceman GA, Karpas Z, Hill HH (2013). Ion mobility spectrometry.

[2] Yousef A, Shrestha S, Viehland LA, Lee EPF, Gray BR, Ayles VL, et al. Interaction potentials and transport properties of coinage metal cations in rare gases. J Chem Phys. 2007. 10.1063/1.2774977. 10.1063/1.2774977 17949151

[3] Mason EA, McDaniel EW (1988). Transport properties of ions in gases.

[4] Kirk AT, Allers M, Cochems P, Langejürgen J, Zimmermann S. A compact high resolution ion mobility spectrometer for fast trace gas analysis. Analyst. 2013. 10.1039/c3an00231d. 10.1039/c3an00231d 23678483

[5] Eiceman GA, Stone JA. Peer reviewed: ion mobility spectrometers in national defense. Anal Chem. 2004. 10.1021/ac041665c. 10.1021/ac041665c 15551477

[6] Borsdorf H, Baldeweg S, Löper F, Höhnisch M, Petrich R, Mayer T. The correlation of odors in the environment with ion mobility spectra patterns. Int J Ion Mobil Spectrom. 2015. 10.1007/s12127-014-0161-9.

[7] Eiceman GA, Blyth DA, Shoff DB, Snyder AP. Screening of solid commercial pharmaceuticals using ion mobility spectrometry. Anal Chem. 2002. 10.1021/ac00213a005. 10.1021/ac00213a005 2382838

[8] Karpas Z. Applications of ion mobility spectrometry (IMS) in the field of foodomics. Food Res Int. 2013. 10.1016/j.foodres.2012.11.029.

[9] Hernández-Mesa M, Escourrou A, Monteau F, Le Bizec B, Dervilly-Pinel G. Current applications and perspectives of ion mobility spectrometry to answer chemical food safety issues. TrAC Trends Anal Chem. 2017. 10.1016/j.trac.2017.07.006.

[10] Arnanthigo Y, Anttalainen O, Safaei Z, Sillanpää M. Sniff-testing for indoor air contaminants from new buildings environment detecting by aspiration-type ion mobility spectrometry. Int J Ion Mobil Spectrom. 2016. 10.1007/s12127-016-0189-0.

[11] Keller T, Keller A, Tutsch-Bauer E, Monticelli F. Application of ion mobility spectrometry in cases of forensic interest. Forensic Sci Int. 2006. 10.1016/j.forsciint.2006.03.032. 10.1016/j.forsciint.2006.03.032 16831529

[12] Midey AJ, Camacho A, Sampathkumaran J, Krueger CA, Osgood MA, Wu C. High-performance ion mobility spectrometry with direct electrospray ionization (ESI-HPIMS) for the detection of additives and contaminants in food. Anal Chim Acta. 2013. 10.1016/j.aca.2013.10.010. 10.1016/j.aca.2013.10.010 24267082

[13] Pfeifer KB, Sanchez RC. Miniaturized ion mobility spectrometer system for explosives and contraband detection. Int J Ion Mobil Spectrom. 2002;5:63–6.

[14] Pfeifer KB, Rohde SB, Peterson KA, Rumpf AN. Development of rolled miniature drift tubes using low temperature co-fired ceramics (LTCC). Int J Ion Mobil Spectrom. 2004;7:52–8.

[15] Babis JS, Sperline RP, Knight AK, Jones DA, Gresham CA, Denton MB. Performance evaluation of a miniature ion mobility spectrometer drift cell for application in hand-held explosives detection ion mobility spectrometers. Anal Bioanal Chem. 2009. 10.1007/s00216-009-2818-5. 10.1007/s00216-009-2818-5 19424683

[16] Ahrens A, Möhle J, Hitzemann M, Zimmermann S. Novel ion drift tube for high-performance ion mobility spectrometers based on a composite material. Int J Ion Mobil Spectrom. 2020. 10.1007/s12127-020-00265-0.

[17] Xu J, William B, Whitten J, Ramsey M. A miniature ion mobility spectrometer. Int J Ion Mobil Spectrom. 2002;5:207–14.

[18] Cook GW, LaPuma PT, Hook GL, Eckenrode BA. Using gas chromatography with ion mobility spectrometry to resolve explosive compounds in the presence of interferents. J Forensic Sci. 2010. 10.1111/j.1556-4029.2010.01522.x. 10.1111/j.1556-4029.2010.01522.x 20735708

[19] Kanu AB, Hill HH. Ion mobility spectrometry detection for gas chromatography. J Chromagr A. 2008. 10.1016/j.chroma.2007.10.110. 10.1016/j.chroma.2007.10.110 18067900

[20] Cohen MJ, Karasek FW. Plasma chromatography—a new dimension for gas chromatography and mass spectrometry. J Chromatogr Sci. 1970. 10.1093/chromsci/8.6.330.

[21] Snyder AP, Harden CS, Brittain AH, Kim MG, Arnold NS, Meuzelaar HLC. Portable hand-held gas chromatography/ion mobility spectrometry device. Anal Chem. 1993. 10.1021/ac00051a019.

[22] Turner RB, Brokenshire JL. Hand-held ion mobility spectrometers. TrAC Trends Anal Chem. 1994. 10.1016/0165-9936(94)87064-0.

[23] Eiceman GA, Feng Y. Limits of separation of a multi-capillary column with mixtures of volatile organic compounds for a flame ionization detector and a differential mobility detector. J Chromagr A. 2009. 10.1016/j.chroma.2008.11.091. 10.1016/j.chroma.2008.11.091 19118835

[24] Ahrens A, Hitzemann M, Zimmermann S. Miniaturized high-performance drift tube ion mobility spectrometer. Int J Ion Mobil Spectrom. 2019. 10.1007/s12127-019-00248-w. 10.1021/acs.analchem.2c03422 PMC9647701 36301910

